# Complications after non-surgical management of proximal humeral fractures: a systematic review of terms and definitions

**DOI:** 10.1186/s12891-019-2459-6

**Published:** 2019-02-23

**Authors:** Stig Brorson, Nikola Alispahic, Christian Bahrs, Alexander Joeris, Amir Steinitz, Laurent Audigé

**Affiliations:** 1 Department of Orthopaedic Surgery, Zealand University Hospital, Køge, Denmark; 2 Department of Clinical Medicine, University of Copenhagen, Copenhagen, Denmark; 3 Department of Orthopaedic Surgery and Traumatology, University Hospital of Basel, Basel, Switzerland; 4 Department of Traumatology and Reconstructive Surgery, Eberhard Karls University Tübingen, BG Trauma Center Tübingen, Tübingen, Germany; 5 AO Clinical Investigation and Documentation, Dübendorf, Switzerland; 6 Crossklinik, Basel, Switzerland; 7 Research and Development Department, Upper Extremities, Schulthess Clinic, Zurich, Institute for Clinical Epidemiology and Biostatistics, University Hospital of Basel, Basel, Switzerland

**Keywords:** Proximal Humeral fractures, Non-surgical, Complications, Adverse events, Systematic review

## Abstract

**Background:**

A majority of proximal humeral fractures can be managed without surgery. Recent randomized clinical trials and meta-analyses even question the benefit of surgical treatment for displaced 3-, and 4-part fractures. However, evidence-based treatment recommendations, balancing benefits and harms, presuppose a common reporting of complications and adverse events, which at the moment is largely missing. Therefore we systematically reviewed the use of terms and definitions of complications after nonsurgical management of proximal humeral fractures.

**Methods:**

We searched PubMed, EMBASE, Cochrane Library, Scopus and WorldCat (2010–2017) and included articles and book chapters containing complication terms or definitions. Two reviewers independently extracted and grouped terms and definitions according to a predefined scheme. Terms and definitions concerning non-surgical management were tabulated, grouped and analyzed qualitatively.

**Results:**

The initial search identified 1376 references from which 470 articles were selected for full-text retrieval. Data-extraction included first articles published in 2017, was then performed iteratively in batches of 20 articles, and terminated after retrieval of 91 articles when no additional definitions or terms was found. In addition, 12 book chapters were reviewed from an initial list of 100. No general definition of a complication was found. A total of 69 terms for complications after non-surgical management were identified from 19 articles. Sixty-seven terms regarded local events. The most commonly reported event terms regarded osteonecrosis, malunion, secondary displacement and rotator cuff problems. Seven individual terms were accompanied by some kind of definition. Most terms and definitions were based on radiographical assessments.

**Conclusions:**

We found no consensus in the use of terms and definitions of complications after nonsurgical management of proximal humeral fractures. Multiple terms, some synonymous, some partly synonymous, some distinct, were used. Few complication terms were explicitly defined. Development and validation of an internationally consensus-based core event set for complications after proximal humeral fractures managed non-surgically is needed.

**Electronic supplementary material:**

The online version of this article (10.1186/s12891-019-2459-6) contains supplementary material, which is available to authorized users.

## Background

Proximal humeral fractures (PHF) are common fractures and account for 4–6% of all fractures [1–3]. They are associated with osteoporosis and 78% of the fractures are seen in patients above the age of 65 [4]. Since 1970 it has been widely believed that 85% of all PHF were minimally displaced and could be managed non-surgically while the remaining 15% were displaced and should be managed surgically [5]. However, more recent epidemiological studies have consistently reported much higher prevalences of displaced fractures ranging from 51 to 86% [3, 6–8]. The most commonly performed surgical procedures include internal fixation with locking plates or humeral nails or replacement of the humeral head with a hemiarthroplasty or a total reverse prosthesis. However, recent randomized clinical trials [9–12] and meta-analyses of randomized trials [13–18] or non-randomized trials [19, 20] have questioned the benefits of these procedures, even for displaced fractures. A call for more non-surgical treatments of PHF has emerged in the scientific literature [21–24].

Any evidence-based recommendation of a treatment modality, surgical or non-surgical, presupposes knowledge on benefits and harms. Guidelines for reporting of clinical effects with validated clinical outcome instruments are available and widely used. However, when it comes to reporting of complications and adverse events after management of PHF there is a paucity of standardized and validated terms and definitions. The majority of clinical studies on PHF deal with surgical management [25] and some complications like hardware failure and infection are obviously linked to surgery. However, complications following non-surgical management of PHF have not been systematically reviewed. Therefore, we aimed to systematically review the use of terms and definitions of complications after non-surgical management of PHF.

## Methods

We conducted a systematic review of published peer-reviewed articles and book chapters according to the PRISMA (Preferred Reporting Items for Systematic Reviews and Meta-Analyses) guidelines [26].

### Search strategy

A search was conducted (June 2017) in PubMed, EMBASE, Cochrane Library and Scopus covering the years 2010–2017. The search strategy for journal articles is found in Additional file 1. For book chapters we searched WorldCat (2016–2017) using the search terms (humer* fra?tur* OR shoulder fra?tu*). We included references in English, German and French language.

### Study selection and data-extraction

After exclusion of duplicates, two reviewers (A.S. and N.A.) screened the initial reference list by title and abstract. A third author (L.A.) reviewed any ambiguous abstracts to reach consensus on the article’s inclusion. Considering all included references we started full-text review and data extraction with the most recent references published in 2017 followed by consecutive series of 20 randomly selected references within previous years in reverse chronology. This process was terminated when all reviewers agreed that no additional relevant information was obtained. For all included references we documented bibliographical data and noted any general definition of ‘complication’ or ‘adverse event’ and any definition of individual complications or adverse events. We documented all individual complication terms reported and grouped them according to the relevant interventions. Terms related to non-surgical interventions were extracted for further analysis. The initial data-extraction was checked by a second reviewer and discrepancies were resolved by consensus. All data were managed and stored in a database using the data capture system REDCap [27] (Version 6.16.5,© 2018 Vanderbilt University).

### Data synthesis

Extracted event terms were organized according to predefined event groups and specifications adapted from Audigé et al. [28]. Event term definitions were tabulated.

## Results

**Fig. 1:**
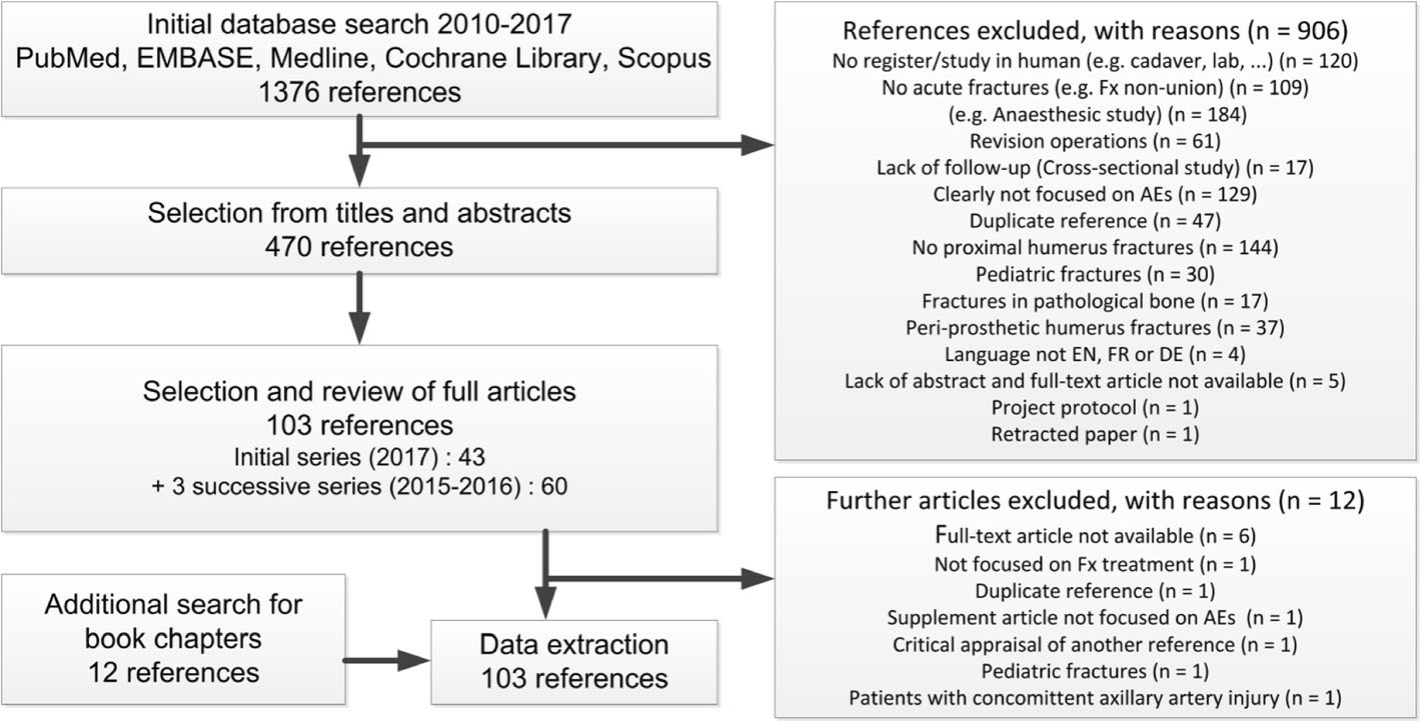
Review flow diagram

The initial search yielded 1376 references (Flow chart, Fig. 1). Based on titles and abstracts we excluded 906 references that did not comply with the inclusion criteria. Thus, 470 references remained for full-text retrieval. Data extraction was terminated in consensus within the review group when 91 articles and 12 book chapters had been retrieved in full text and no new terms or definitions was identified in the last group of references.

A total of 19 references (15 articles [13, 29–42] and 4 book chapters [24, 43–45]) reported terms and definitions of complications after non-operative management of PHF. The remaining references were excluded because they dealt with surgical management exclusively. From all the terms that were documented as being reported in the context of non-operative treatment, we identified the related papers, and then by checking back to these papers found out that only 19 papers were specifically focused on non-operative management.

After excluding spelling errors and clearly synonymous words 69 complication terms remained for further analysis (Table 1). They were grouped into 7 broad groups and 11 subgroups. Seven complication terms were defined.

**Table 1:** Adverse event terms

Event group	Event subgroup	Event term
Osteochondral		Heterotopic bone formation
		Humeral head resorption
	Arthritis	Degenerative arthritis
		Osteoarthritis
		Post-traumatic arthritis
		Post traumatic osteoarthritis
	Tuberosity migration/resorption	Superior migration of greater tuberosity
		Posterior migration of lesser tuberosity
		Medial displacement of the greater tuberosity
		Displaced greater tuberosity
	Osteonecrosis	Osteonecrosis
		Osteonecrosis of the humeral head
		Necrosis of the humeral head
		Avascular necrosis of the humeral head
		Avascular necrosis
		Head avascular necrosis
		Humeral head ischemia
		Loss of perfusion of the humeral head
	Delayed union	Delayed union
		Prolonged delayed union
	Malunion	Malunion
		Valgus malunion
		Varus malunion
		Varus malunion in anteversion
		Varus malunion in retroversion
		Greater tuberosity malunion
		Malunion of the tuberosities
	Nonunion	Nonunion
		Fracture non-union
		Pseudoarthrosis
	Secondary fracture displacement	Secondary displacement
		Fracture displacement
		Varus collapse
		Cephalic collapse
		Complete displacement of the humeral shaft
		Malposition of lesser tuberosity
		Malreduction^a^
		Poor fracture reduction^a^
Instability		Recurrent shoulder dislocation
Shoulder pain		Pain
		Persistent pain
		Shoulder pain
Neurological		Iatrogenic neurovascular injury^a^
		Axillary nerve lesions
		Complex regional pain syndrome
Soft tissue (superficial)		Skin irritation
Soft tissue (deep)	Impingement	Impingement
		Impingement of the greater tuberosity
		Subacromial impingement
		Internal rotation impingement
		Coracoidal impingement
		Impingement of the greater tuberosity on the acromion
	Capsular	Capsular contracture
		Capsulitis
		Adhesive capsulitis
		Secondary adhesive capsulitis
		Frozen shoulder
	Stiffness	Stiffness
		Shoulder stiffness
		Stiff shoulder
		Self-limiting stiffness
	Rotator cuff	Rotator cuff tear
		Rotator cuff pain
		Rotator cuff weakness
		Rotator cuff deficiency
		Rotator cuff dysfunction
		Rotator cuff injury
Non-local		Pneumonia
		Deep Venous Thrombosis

^a^After closed reduction

### Complication terms

All 69 complication terms were initially divided into local and non-local events. Local events were further grouped into ‘osteochondral’, ‘instability’, ‘shoulder pain’, ‘neurological’, ‘soft tissue (superficial)’, and ‘soft tissue (deep)’.

The largest group (39 terms) was the ‘osteochondral’ group covering the subgroups ‘arthritis’, ‘tuberosity migration/resorption’, ‘osteonecrosis’, ‘delayed union’, ‘malunion’ and ‘secondary fracture displacement’. All 39 event terms in this group were radiographically based.

The second largest group, ‘soft tissue (deep)’ (21 terms) covered ‘impingement’, ‘capsular’, ‘stiffness’ and ‘rotator cuff’. These event terms were defined clinically or by magnetic resonance imaging (MRI).

The remaining event terms were related to instability, pain, neurological injury, skin problems and the non-local events pneumonia and deep venous thrombosis.

### Definitions

Among the full text searches we found 7 complication definitions. Six out of 7 definitions regarded radiographically defined events like malunion, nonunion, displacement and avascular necrosis (Table 2). Loss of power in arm was the only non-radiographically defined event term.

**Table 2:** Summary of definitions of adverse events

Author	Event term	Event definition
Handoll (2015) [13]	Avascular necrosis (score 2–0)	Score 2 = no changes/1 = changes to normal trabecular organisation < 50% of humeral head/0 = > 50% of humeral head or partial collapse
Handoll (2015) [13]	Secondary varus displacement	> 10°
Kancherla (2017) [39]	Tuberosity displacement	> 5 mm.
Fang (2017) [29]	Loss of power in arm (grade 0–5)	Medical Research Council Scale (grade 0–5) [47]
Papakonstantinou (2017) [41]	Delayed union/non-union/prolonged delayed union	Union between 61 and 89 days/when fractures had not united by 90 days/union after 90 days
Papakonstantinou (2017) [41]	Varus malunion	Neck-shaft angle ≤110°
Papakonstantinou (2017) [41]	Non-union (indirect definition)	Presence of callus uniting the main fragments of fractures in 3 of the 4 bone cortices

## Discussion

We found no consensus in the use of terms and definitions of complications after non-surgical management of PHF. Only very few definitions of complications and adverse events were identified. Relatively few references on non-surgical management were identified compared to surgical interventions. This confirms the findings of Slobogean et al. [25] who conducted a scoping review of the literature on PHF and reported that less than 5% of the body of literature dealt with non-surgical management compared to more than two thirds concerning surgical management. Despite this bias towards surgical literature we find it important to focus on complications after non-surgical management. A systematic reporting of complications and adverse events is needed for evidence-based suggestions and balanced decision-making [46].

### ‘Radiographical complications’

Most terms and definitions of adverse events are based on assessments of radiographs. Assessments based on radiographs may favor surgical management as osteosynthesis and arthroplasty aim to restore the anatomy of the proximal humerus or to replace the damaged joint. To designate a certain radiographic pattern as a complication or an adverse event does not necessarily mirror the functional outcome and expectations as reported by the patient. Displaced fractures in adults can be expected to heal with some degree of malunion when treated non-surgically. In that sense, a malunion is not necessarily an adverse event from the patient’s perspective. Even severe maluninon may be tolerated by patients with limited functional demands. More knowledge is needed to clarify the association between patient reported outcome and radiographically defined complications after non-surgical management.

Displacement, migration, malunion and nonunion are continuous variables brought into distinct categories often by poorly defined cut-off values. Three references proposed explicit definitions of ‘secondary varus displacement’ [13] ‘tuberosity displacement’ [39] and ‘varus malunion’ [41] based on measurements of degrees and millimeters on radiographs. The scientific and clinical validity of such definitions may be questioned and further studies may contribute to elucidate the clinical relevance of these commonly used complication terms.

The complication terms and definitions identified for non-surgical management can roughly be divided generically into three groups:

### Pathoanatomical entities

‘Humeral head necrosis’ and ‘capsulitis’ are pathoanatomical diagnoses applied to radiological, clinical or intra-operative findings. Similarly, non-local terms like ‘pneumonia’ and ‘DVT’ are clinical and para-clinical (radiographs, ultrasound, blood tests) diagnoses rarely verified by pathologists.

### Pathophysiological entities

‘Loss of perfusion’ leading to ‘humeral head ischemia’ and eventually ‘avascular necrosis of the humeral head’ are successive changes in a pathophysiological process. This process is quantified in the 3-stage definition of ‘avascular necrosis’ [13].

The process leading to ‘non-union’ or ‘pseudoarthrosis’ is captured in the 3-stage definition ‘delayed union’, non-union’, or ‘prolonged delayed union’ [41].

### Biomechanical entities

The terms related to rotator cuff problems are based on a biomechanical understanding of successive changes caused by muscular imbalance. The term ‘rotator cuff’ is usually followed by specifications like ‘tear’ and ‘injury’ (based on imaging), ‘pain’ (based on history), or ‘dysfunction’ and ‘deficiency’ (based on a functional understanding).

The terms related to ‘impingement’ are based on a biomechanical understanding of the process leading to pain and impairment. ‘Internal rotation impingement’ is clinically defined while ‘impingement of the greater tuberosity on the acromion’ illustrates a biomechanical understanding.

### Future aspects

To obtain consensus on terms and definitions we plan to apply a Delphi consensus process based on the findings from the systematic review. An international group of shoulder surgeons will independently assess and comment on the proposed terms and definitions through a series of online surveys. A core event set will be developed and further validated. A similar approach has previously been applied to complications associated with arthroscopic rotator cuff tear repair [28].

## Conclusions

Based on this systematic review we found no consensus on terms and definitions of complications and adverse events after non-surgical management of PHF. Most terms and definitions are based on radiographical assessments and the clinical relevance of terms and definitions from the patients’ perspective remains to be demonstrated. We recommend steps towards the development of a core event set of complication terms based on consensus among shoulder and trauma specialists and with involvement of patient representatives in the validation process.

## Additional file

Additional file 1: Search protocol for proximal humeral fractures in PubMed, Medline, Embase, Cochrane Library and Scopus. (PDF 649 kb)

## Abbreviations

DVT: Deep venous thrombosis
MRI: Magnetic resonance imaging
PHF: Proximal humeral fracture

## References

[1] Buhr AJ, Cooke AM (1959). Fracture patterns. Lancet.

[2] Knowelden J, Buhr AJ, Dunbar O (1964). Incidence of fractures in persons over 35 years of age. A report to the M.R.C. Working party on fractures in the elderly. Br J Prev Soc Med.

[3] Court-Brown CM, Garg A, McQueen MM (2001). The epidemiology of proximal humeral fractures. Acta Orthop Scand.

[4] Olsson C, Nordqvist A, Petersson CJ (2004). Increased fragility in patients with fracture of the proximal humerus: a case control study. Bone.

[5] Neer CS (1970). Displaced Proximal Humeral fractures. J Bone Jt Surg.

[6] Roux A, Decroocq L, El Batti S, Bonnevialle N, Moineau G, Trojani C (2012). Epidemiology of proximal humerus fractures managed in a trauma center. Orthopaedics and Traumatology: Surgery and Research.

[7] Tamai K, Ishige N, Kuroda S, Ohno W, Itoh H, Hashiguchi H (2009). Four-segment classification of proximal humeral fractures revisited: a multicenter study on 509 cases. J Shoulder Elb Surg.

[8] Bahrs C, Stojicevic T, Blumenstock G, Brorson S, Badke A, Stöckle U (2014). Trends in epidemiology and patho-anatomical pattern of proximal humeral fractures. Int Orthop.

[9] Olerud P, Ahrengart L, Ponzer S, Saving J, Tidermark J (2011). Hemiarthroplasty versus nonoperative treatment of displaced 4-part proximal humeral fractures in elderly patients: a randomized controlled trial. J Shoulder Elb Surg.

[10] Rangan A, Handoll H, Brealey S, Jefferson L, Keding A, Martin BC (2015). Surgical vs nonsurgical treatment of adults with displaced fractures of the proximal humerus the PROFHER randomized clinical trial. JAMA -J Am Med Assoc.

[11] Boons HW, Goosen JH, Van Grinsven S, Van Susante JL, Van Loon CJ (2012). Hemiarthroplasty for humeral four-part fractures for patients 65 years and older a randomized controlled trial. Clin Orthop Relat Res.

[12] Fjalestad T, Hole M, Jørgensen JJ, Strømsøe K, Kristiansen IS (2010). Health and cost consequences of surgical versus conservative treatment for a comminuted proximal humeral fracture in elderly patients. Injury.

[13] Handoll HH, Brorson S. Interventions for treating proximal humeral fractures in adults. Cochrane Database Syst Rev. 2015;(11):11, CD000434. 10.1002/14651858.CD000434.pub4 26560014

[14] Xie L, Ding F, Zhao Z, Chen Y, Xing D (2015). Operative versus non-operative treatment in complex proximal humeral fractures: a meta-analysis of randomized controlled trials. Springerplus.

[15] Mao Z, Zhang L, Zhang L, Zeng X, Chen S, Liu D (2014). Operative versus nonoperative treatment in complex Proximal Humeral fractures. Orthopedics.

[16] Rabi S (2015). Operative *vs* non-operative management of displaced proximal humeral fractures in the elderly: a systematic review and meta-analysis of randomized controlled trials. World J Orthop.

[17] Fu T, Xia C, Li Z (2014). Wu H. Surgical versus conservative treatment for displaced proximal humeral fractures in elderly patients: a meta-analysis. Int J Clin Exp Med.

[18] Xie L, Ding F, Zhao Z, Chen Y, Xing D (2014). Operative versus non-operative treatment in complex proximal humeral fractures: a meta-analysis of randomized controlled trials. Orthopedics.

[19] den HD, de HJ, Schep NW, Tuinebreijer WE (2010). Primary shoulder arthroplasty versus conservative treatment for comminuted Proximal Humeral fractures: a systematic literature review. Open Orthop J.

[20] Beks RB, Ochen Y, Frima H, Smeeing DPJ, van der Meijden O, Timmers TK (2018). Operative versus nonoperative treatment of proximal humeral fractures: a systematic review, meta-analysis, and comparison of observational studies and randomized controlled trials. J Shoulder Elb Surg.

[21] Maffulli N (2017). We are operating too much. J Orthop Traumatol.

[22] Aspenberg P (2015). Why do we operate proximal humeral fractures?. Acta Orthop.

[23] Jefferson L, Brealey S, Handoll H, Keding A, Kottam L, Sbizzera I (2017). Impact of the PROFHER trial findings on surgeons’ clinical practice. Bone Jt Res.

[24] Brorson S, Court-Brown C, McQueen MM, Swiontkowski MF, Ring D, Friedman SM, Duckworth A (2017). Proximal Humeral Fractures. Musculoskeletal trauma in the elderly. Taylor & Francis Group.

[25] Slobogean GP, Johal H, Lefaivre KA, MacIntyre NJ, Sprague S, Scott T, et al. A scoping review of the proximal humerus fracture literature orthopedics and biomechanics. BMC Musculoskelet Disord. 2015;16. 10.1186/s12891-015-0564-8 PMC4464621 25958203

[26] Preferred Reporting Items for Systematic Reviews and Meta-Analyses (PRISMA), http://www.prisma-statement.org

[27] Harris PA, Taylor R, Thielke R, Payne J, Gonzalez N, Conde JG (2009). Research electronic data capture (REDCap)-a metadata-driven methodology and workflow process for providing translational research informatics support. J Biomed Inform.

[28] Audigé L, Flury M, Müller AM, Durchholz H (2016). Complications associated with arthroscopic rotator cuff tear repair: definition of a core event set by Delphi consensus process. J Shoulder Elb Surg.

[29] Fang C, Kwek EBK. Self-reducing proximal humerus fractures. J Orthop Surg (Hong Kong). 2017;25. 10.1177/2309499017717180 28659054

[30] Nikola C, Hrvoje K, Nenad M (2014). Reverse shoulder arthroplasty in acute fractures provides better results than in revision procedures for fracture sequelae. Int Orthop.

[31] Okike K, Lee OC, Makanji H, Morgan JH, Harris MB, Vrahas MS (2015). An Original Study Comparison of Locked Plate Fixation and Nonoperative Management for Displaced Proximal Humerus Fractures in Elderly Patients.

[32] Patel S, Colaco HB, Elvey ME, Lee MH (2015). Post-traumatic osteonecrosis of the proximal humerus. Injury.

[33] Schairer WW, Nwachukwu BU, Lyman S, Craig EV, Gulotta LV (2015). Reverse shoulder arthroplasty versus hemiarthroplasty for treatment of proximal humerus fractures. J Shoulder Elb Surg.

[34] Sabharwal S, Patel NK, Griffiths D, Athanasiou T, Gupte CM, Reilly P (2016). Trials based on specific fracture configuration and surgical procedures likely to be more relevant for decision making in the management of fractures of the proximal humerus: findings of a meta-analysis. Bone Joint Res.

[35] Sethi PM, Macken CJ (2016). Management of Greater Tuberosity Fractures. Tech Shoulder Elb Surg.

[36] Sharaby MMF (2016). Results of biological restoration of varus impacted proximal humeral fracture and stabilization with locked plate and calcar screws. Curr Orthop Pract.

[37] Biazzo A, Cardile C, Brunelli L, Ragni P, Clementi D (2017). Early results for treatment of two- and three-part fractures of the proximal humerus using contours PHP (proximal humeral plate). Acta Biomed.

[38] Hageman MGJS, Meijer D, A. Stufkens S, Ring D, N. Doornberg J, Ph. Steller E. Proximal Humeral Fractures: nonoperative versus operative treatment. Arch Trauma Res 2016;6.

[39] Kancherla VK, Singh A, Anakwenze OA (2017). Management of Acute Proximal Humeral Fractures. J Am Acad Orthop Surg.

[40] Lowry V, Bureau NJ, Desmeules F, Roy JS, Rouleau DM (2017). Acute proximal humeral fractures in adults. J Hand Ther.

[41] Papakonstantinou MK, Hart MJ, Farrugia R, Gosling C, Kamali Moaveni A, van Bavel D (2017). Prevalence of non-union and delayed union in proximal humeral fractures. ANZ J Surg.

[42] Park YK, Kim SH, Oh JH (2017). Intermediate-term outcome of hemiarthroplasty for comminuted proximal humerus fractures. J Shoulder Elb Surg.

[43] Brunner UH. Kapitel 18 - Kopferhaltende Therapie der proximalen Humerusfraktur. In: Habermeyer P, Lichtenberg S, Loew M, Magosch P, Martetschläger F, Tauber M, editors. Schulterchirurgie (Fünfte Ausgabe). Fünfte Aus. Munich: Urban: Fischer; 2017. p. 483–534.

[44] Esenyel EZ (2017). The shoulder. The shoulder. Cham: springer international publishing, Cham.

[45] Bohsali K, ABMW (2017). Fractures of the Proximal Humerus. Rockwood and Matsen’s the shoulder. Fifth edit.

[46] Jacxsens M, Walz T, Durchholz H, Müller AM, Flury M, Schwyzer HK, Audigé L (2017). Towards standardised definitions of shoulder arthroplasty complications: a systematic review of terms and definitions. Arch Orthop Trauma Surg.

[47] John J (1984). Grading of muscle power: comparison of MRC and analogue scales by physiotherapists. Medical Research Council Int J Rehabil Res Int Zeitschrift fur Rehabil Rev Int Rech Readapt.

